# Quality of web-based Arabic health information on dental implants: an infodemiological study

**DOI:** 10.1186/s12903-023-02938-8

**Published:** 2023-04-20

**Authors:** Esam Halboub, Mohammed Sultan Al-Ak’hali, Ahmed Shaher Alqahtani, Ehab A. Abdulghani, Mona Awad Kamil, Mohammed Nasser Alhajj, Abeer A. Al-Sosowa

**Affiliations:** 1 Department of Maxillofacial Surgery and Diagnostic Sciences, College of Dentistry, Jazan University, Jazan, Saudi Arabia; 2 Department of Oral Medicine, Oral Pathology and Oral Radiology, Faculty of Dentistry, Sana’a University, Sana’a, Yemen; 3 Department of Preventive Dental Sciences, College of Dentistry, Jazan University, Jazan, Saudi Arabia; 4 Department of Periodontology, Faculty of Dentistry, Sana’a University, Sana’a, Yemen; 5 Department of Orthodontics and Dentofacial Orthopedics, College of Dentistry, West China Hospital of Stomatology, Sichuan University, Chengdu, China; 6 Department of Orthodontics and Dentofacial Orthopedics, College of Dentistry, Thamar University, Dhamar, Yemen; 7 Department of Prosthodontics, Faculty of Dentistry, Thamar University, Dhamar, Yemen; 8 Department of Periodontics, Faculty of Dentistry, Thamar University, Dhamar, Yemen

**Keywords:** Dental implant, Health information, Infodemiology, Internet-based information, Quality Assessment, Readability

## Abstract

**Background:**

In the era of the internet, patients seek health information ahead of getting the required treatment. Dental implant, which is among the most sought dental treatments, is not an exception. Incorrect health related information may lead to harmful deeds, so this study sought to assess the quality of web-based Arabic health information on dental implants.

**Methods:**

The following engines were searched: Google (http://www.google.com), Yahoo! (http://www.yahoo.com), and Bing (http://www.bing.com) on 13 January 2022 for specific Arabic terms on “dental implants”. The first 100 consecutive websites from each engine were analyzed for eligibility. The eligible websites were assessed using JAMA benchmarks tool, DISCERN tool, and HONcode. An online tool (including FKGL, SMOG and FRE) was used to assess readability of the websites.

**Results:**

There were 65 eligible websites, of which only one (1.5%) was HONcode certified. Only 3 (4.5%) websites attained a high score (> 65 out of 80) based on DISCERN tool: The mean DISCERN score was 41.14 ± 12.64. The mean JAMA score was 1.69 ± 1.13; however, only five (7.6%) met all JAMA criteria. The main shortcomings were attributed to not meeting the “Attribution” (54 [83.1%]) and “Authorship” (43 [66.2%]) criteria. The mean grade level of FKGL score was 7.0 ± 4.5. The majority of the websites (60%) scored less than 7, indicating easy content to understand. The mean grade level of SMOG score required to understand a website’s text was 3.2 ± 0.6. Around 91% of the websites had reading ease scores ≥ 80, suggesting that the website’s content was easy to read.

**Conclusion:**

Unfortunately, although readable, most of the easily accessible web-based Arabic health information on dental implants does not meet the recognized quality standards.

**Supplementary Information:**

The online version contains supplementary material available at 10.1186/s12903-023-02938-8.

## Introduction

Nowadays, dental implants are the most dental prosthesis sought for tooth/teeth replacement owing to the fact they are designed to look and function like the natural teeth. Although it requires inter-professional teamwork including periodontist, surgeon, restorative dentist and laboratory, dental implant has many advantages when compared with other alternative treatments (dentures, dental bridges, crowns, and others). Among these advantages are the high success rate, longevity, bone maintenance, less plaque retention, less risk of exposing adjacent teeth to caries, endodontic problems and teeth sensitivity, and high patient’s satisfaction.

The patients are aware about various treatment choices, and dental implants are not an exception. They will not accept any treatment unless they are saturated with adequate knowledge about all important information [1]. Previously, the health professionals were the source of such information. The widespread use of the internet also increases its use for obtaining health-related information. The use of health-related websites has increased in parallel with this change. It has been observed that health sites give information with the intention of providing diagnostic and therapeutic services [2]. Murray et al. [3] established that 85% of physicians reported a patient bringing internet information ahead of the planned visit. In the era of the internet, all ask “Google”. Unfortunately, the internet frequently provides enormous amount of suspicious, irrelevant, doubtful, and even fake information [4]. This is driven by the strong marketing atmosphere and commercials promotion which focus on the attractive side of a given health care service like dental implants, as it is the case with other services or products [5, 6].

Routinely, dental personnel are questioned about dental information seen in the internet. Dental implant is one of the most common dental topics the patients used to ask about. Given such an eagerness to learn about dental implant, dental professionals must direct their patients to the reliable, readable evidence based websites. Unfortunately, this is not the case: Many studies reported that the dental implant material in the internet are unreadable and even beyond the understanding capability of the targeted readers [1].

Incorrect health related information may lead to harmful deeds such as using unlicensed remedies, toxic herbs, and wrong prophylactic strategies. So it is mandatory that these websites be assessed for the quality of information they publish [7–12]. In this context, one recent study found that the available web-based health information on dental implants in English language is difficult to read for the average patient, in addition to being poor in terms of quality [13]. With such information, dental professionals will find it difficult to convince dental patients with the real information, leading ultimately to conflicted patient-dentist relationship. This unfortunately applies across different medical/dental disciplines. Indeed, in the era of the internet, patients think it is easy to diagnose and/or treat their selves, despite the level of reliability of the information they come across in the internet. At the best, the patients flick through a huge number of websites seeking for the signs and symptoms of their already diagnosed diseases, with a especial focus on the complications and prognosis. The matter of searching, filtering, and choosing reliable information is not an easy task, more specifically in the era of the internet. This applies even for those who are highly educated and acknowledged, like physicians and dentists [14]. That is why many organizations and authorities stress on applying the evidence-based dental/medical practices. In this context, we know the effort researchers exert in conducting a systematic review to summarize an evidence on one intervention or association. In doing so, they always come across many irrelevant studies and exclude them due to being irrelevant or not fitting the inclusion criteria, although these excluded studies are found in trusted search engines and/or databases. The situation is completely different when we talk about the patients; they basically search everywhere in the internet, and don’t apply pre-defined criteria in order to dispute a piece of health information [15]. Unfortunately, they mostly trust the information they come across in the internet. Imagine this tragedy is in the context of the information in the English websites! How will the situation be in the context of other, less common and used languages?

Regarding the web-based Arabic health information, a few studies have been conducted addressing oral cancer, COVID-19, denture hygiene, and periodontal diseases, and revealed low quality [7–10]. To our best knowledge, no single study has been done so far to evaluate the quality and readability of web-based Arabic health information on dental implant. We hypothesized that the web-based Arabic information on the dental implant are of high quality and readable. Hence, this study aimed to assess the quality and readability of web-based Arabic health information regarding dental implant.

## Methods

### Search strategy

**Fig. 1:**
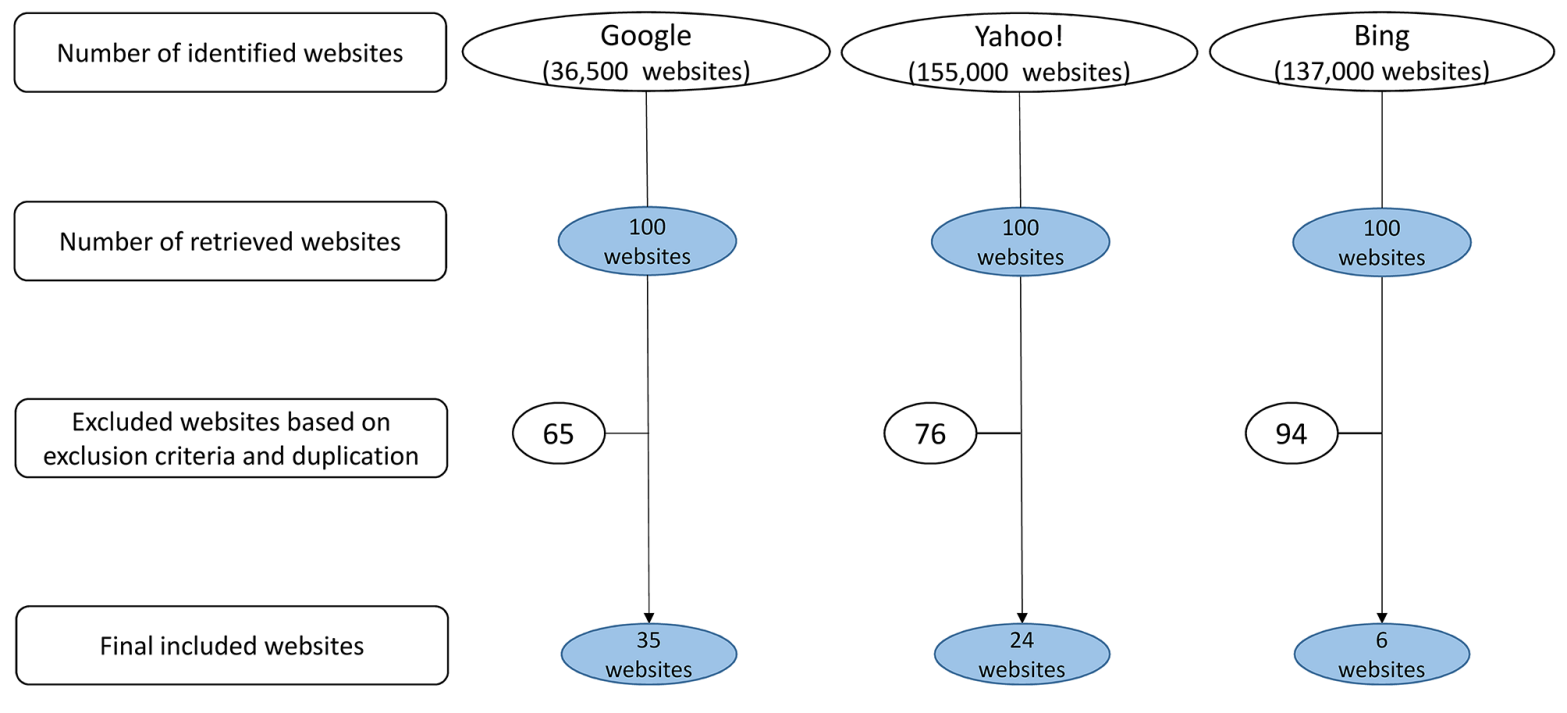
Flow chart of the search strategy

We searched, using Google Chrome, version 81.0.4044, the following engines: Google (http://www.google.com), Yahoo! (http://www.yahoo.com), and Bing (http://www.bing.com) on 13 January 2022, following “The Pew Research Center’s Internet & American Life Project”[16], which confirmed that up to 79% of online health seekers use one of these search engines. Cookie information was erased ahead of browsing and searching. In addition, we browsed using “incognito” (private) mode, in order to prevent any biases that could arise from preceding searches [7]. We used the agreed-upon Arabic translations of the following most widely used English terms describing dental implant as search keywords: “dental implant”, “dental implants” “tooth implant”, and “teeth implants”. The first 10 consecutive pages (the first 100 consecutive websites) from each engine were included [9]. Two authors (EH and MSA) independently checked the duplicates, and when present were removed. A given website was excluded if: 1) non-Arabic language; 2) information presented as hints or exclusively audio- or video-wise; 3) being scientific articles or textbooks; 4) presence of banner advertisements or sponsored links and discussion forums; 5) sites that were blocked or denied direct access (required ID and password); and 6) being social forums and/or social media websites [10]. Then, the relevant websites presenting health information about dental implant in the Arabic language were selected and evaluated for quality and readability analyses. The different stages of the search strategy that we followed are depicted in Fig. 1.

### Quality assessment tools

The quality of the included websites was assessed using the following tools: the Health on the Net Foundation Code of Conduct (HONcode) [17], DISCERN tool [18], and JAMA benchmarks [19]. With regard to the HONcode tool, it grants a permission for a given qualified website to display a stamp (HON award-like badge) which is a certificate-like badge that remains valid for 1 year only on the condition that the said website complies with HONcode criteria. The software of HONcode was downloaded and incorporated into Google Chrome as an extension. Accordingly, the HONcode seal appeared only on the certified websites with each search. Moreover, and for further confirmation, the websites with the HONcode seals were rechecked at the main HONcode website for the currency of its certificate [17].

The JAMA benchmarks tool, which is published by the Journal of the American Medical Association, evaluates the following criteria: (1) authorship (availability of data on authors, their contributions, affiliations, and relevant credentials); (2) attribution (mentioning references and sources from which the content was cited); (3) disclosure (availability of data on ownership, sponsorship, advertising, underwriting, commercial funding or support sources and any potential conflicts of interest); and (4) currency (mentioning clearly the dates of initial posting and updating of the content). Each criterion, when fulfilled (“yes” response) got a score of one point for the website; otherwise, it was scored zero (0) point. Accordingly, the overall JAMA score ranges from 0 where no criteria fulfilled to 4 points where all 4 criteria fulfilled [19].

The DISCERN tool comprised 16 questions included in three main sections: questions 1–8 address the extent of trustfulness of the websites as sources of data with regard to selected therapies, questions 9–15 address therapy alternatives, and question 16 assesses the overall quality score. Each question ranges from 1 to 5, where 1 indicating a poor website and 5 indicating a good quality website. Hence, the minimum score is 16 and the maximum is 80. The obtained scores were categorized as low (16–32), moderate (33–64), and high (≥ 65). Two of the authors (EH and MSA) conducted the quality assessment using the DISCERN and JAMA tools. For calibration, they independently assessed 10 websites and discussed and resolved any discrepancies, if any. Later, inter-examiner agreement was calculated for all of the websites [18].

### Readability assessment

An online calculator tool of readability was used to evaluate the readability of the websites [20]. The Online Utility website indicates that this tool can be used for many languages, however, it was primarily designed to evaluate English language text. This website evaluates the text using well-known, common analytic tools (Gunning Fog Index (GFI), Coleman Liau Index (CLI), Flesch Kincaid grade level (FKGL), Automated Readability Index (ARI), Simple Measure of Gobbledygook (SMOG), and Flesch Reading Ease (FRE)). For the readability assessment of the Arabic text, the SMOG, FRE, and FKGL were selected. The other indices were excluded because they formulate the readability score based on the number of letters. This formula does not apply to Arabic text, because simply the Arabic words, in contrast to English words, are consist of letters that are connected to one another. The acceptable level of readability was set to < 7 for the FKGL and SMOG, and ≥ 80 for the FRE [21, 22].

### Statistical analysis

All statistical data were analyzed by SPSS (Statistical Package for Social Sciences) Version 22.0 software program. The test of normality was utilized using the Shapiro–Wilk test. Spearman correlation coefficient test was used to evaluate the correlation between the indices. A P value < 0.05 was set to be a statistical significance.

## Results

Figure 1 presents the search strategy and its results. The search yielded a total of 328.500 websites from the three engines (36.500 from Google; 155.00 from Yahoo; and 137.000 from Bing). Of the first 300 screened websites, we excluded 147 websites due to being duplicates. The remaining 153 websites were analyzed for eligibility, where 88 websites were excluded due to either not in the Arabic language, presenting irrelevant information, social forums, or presenting audio or video content only. Accordingly, 65 eligible websites (Additional file 1: Supplementary file) were assessed for quality and readability. Interestingly, there was only one (1.5%) website (https://www.mayoclinic.org/ar/tests-procedures/dental-implant-surgery/about/pac-20384622) which was HONcode certified.

As shown in Table 1, only 3 (4.5%) websites attained a high score (> 65 out of 80) based on the criteria of the DISCERN tool: The mean DISCERN score of all websites was 41.14 ± 12.64, ranging from as low as 21 to as high as 69. With regard to the JAMA benchmarks results, a mean score of 1.69 ± 1.13 was achieved by the websites. However, only five (7.7%) met all the JAMA criteria. The main shortcomings regarding JAMA were attributed to not meeting the “Attribution” (54 [83.1%]) and “Authorship” (43 [66.2%]) criteria. In terms of readability, the mean words and sentences numbers were found to be 1324.7 ± 1164.3 and 57.9 ± 61.3, respectively. The mean grade level of the FKGL score was 7.0 ± 4.5. The majority of the websites (60.0%) scored less than 7, indicating that the website’s content was easy for the general public to understand. The mean grade level of the SMOG score required to understand a website’s text was 3.2 ± 0.6. Around 91% of the websites had reading ease scores ≥ 80, suggesting that the website’s content was easy for the general public to read. More details are shown in Table 1.

**Table 1:** Quality assessment of the included websites (n = 65)

Criteria	Frequency	Percent	Mean (SD)	Min - Max
**HONcode**				
Certified	1	1.5		
Not-certified	64	98.5		
**DISCERN**			41.14 (12.64)	21–69
High (≥ 65)	3	4.6		
Moderate (33–64)	38	58.5		
Low (16–32)	24	36.9		
**JAMA Benchmarks**			1.69 (1.13)	0–4
No item met	7	10.8		
One Item met	28	43.1		
Two items met	13	20		
Three items met	12	18.5		
Four items met	5	7.7		
**Authorship-JAMA**				
0 (Not met)	43	66.2		
1 (Met)	22	33.8		
**Attribution-JAMA**				
0 (Not met)	54	83.1		
1 (Met)	11	16.9		
**Disclosure-JAMA**				
0 (Not met)	14	21.5		
1 (Met)	51	78.5		
**Currency**				
0 (Not met)	39	60		
1 (Met)	26	40		
**Number of words**			1324.7 (1164.3)	73–5256
**Number of sentences**			57.9 (61.3)	2–259
**Flesch Kincaid Grade level**			7.0 (4.5)	0.7–27.6
< 7	39	60.0		
≥ 7	26	40.0		
**Simple Measure of Gobbledygook**			3.2 (0.6)	3–5.9
< 7	65	100		
≥ 7	0	0		
**Flesch Reading Ease**			94.2 (11.9)	40.4–110.5
<80	6	9.2		
≥80	59	90.8		

Table 2 presents the associations between readability indices, DISCERN and JAMA. DISCERN had a strong significant positive correlation with the number of words, sentences, and JAMA P < 0.001. A significant, positive correlation was found between the number of words with SMOG (r = 0.280; P = 0.024). The number of sentences had a significant, negative correlation with FKGL (r = − 0.464; P < 0.001), whereas it had a significant, positive correlation with SMOG and FRE (r = 0.257; P = 0.039, r = 0.465; P < 0 0.001), respectively. FKGL had a highly significant negative correlation with the FRE.

**Table 2:** Correlation between the DISCERN, JAMA, and Readability indices

		JAMA	DISCERN	No. words	No. sentences	FKGL	SMOG	FRE
**JAMA**	*rho*	1.000	0.612^**^	0.278^*^	0.241	-0.062	-0.032	0.066
	P*		0.000	0.025	0.053	0.625	0.797	0.602
**DISCERN**	*rho*		1.000	0.648^**^	0.553^**^	-0.020	0.063	0.026
	P*			0.000	0.000	0.876	0.617	0.836
**No. words**	*rho*			1.000	0.910^**^	-0.116	0.280^*^	0.121
	P*				0.000	0.357	0.024	0.339
**No. sentences**	*rho*				1.000	− 0.464^**^	0.257^*^	0.465^**^
	P*					0.000	0.039	0.000
**FKGL**	*rho*					1.000	-0.044	− 0.994^**^
	P*						0.727	0.000
**SMOG**	*rho*						1.000	0.022
	P*							0.862
**FRE**	*rho*							1.000
	P*							

*Spearman correlation coefficient test

## Discussion

This study aimed to investigate the quality and readability of Arabic health information on a very hot issue pertinent to the population: dental implants. To attain our aim, we utilized the most frequently used search engines: Google, Yahoo, and Bing. Indeed, a previous study demonstrated that many patients seek for e-health online information to initiate their quest through one of these search engines [23]. Owing to the relative instability of search engines’ reproducibility, we carried out our extraction of data in one sitting. The first 100 websites (the first 10 pages) were explored across each search engine simply due to that the internet seekers don’t go beyond 10 pages. In addition, we applied strict search criteria [24]. Our results demonstrated that although on average the assessed websites are somewhat readable and hence quite easy to be understood, the quality of their content does not meet recognized quality standards. This complicates the case; imagine the internet users easily understand the faulty health information: it is a disaster.

In this era, most, if not all, patients seek for information on preventive and therapeutic remedies for their diseases in the internet, despite the quality of such information. Unfortunately, not only new internet users but frequent internet users also tend to believe such false information they come across. Indeed, when a patient browses through websites with false information, he/she is not able to identify them as wrong, and believes what he/she read [25]. An earlier study showed that patients are least bothered about the authenticity, content and quality of the information while seeking medical data online [26].

The HONcode, DISCERN, and JAMA Benchmarks tools are the most commonly used in order to assess the reliability and quality of the information available online on medical websites [27–29]. Regrettably, only one website was HONcode certified (mayoclonic.org). Moreover, the content of that website is not Arabic in origin; instead, it is just translation of original English content. The other sixty-four websites either didn’t apply to get the HONcode or applied to get it but didn’t comply with the criteria and hence not approved. In fact, HONcode is a trust mark of the content of a given website, and perhaps the non-certified HONcode websites contain misleading, bad-quality, subjective and vague medical data for the internet users. All in all, HONcode-certified websites are obliged (and be proud) to show the HONcode seal on their websites as this shows the authenticity of the information available. HONcode-certified websites get regular audits and compliance checks [30].

Regrettably too that only three websites achieved a high DISCERN score (≥ 65 out of 80). This represents 4.6% of the health information available online on dental implant in the Arabic language. The majority of the websites attained a moderate (58.5%) and low (36.9%) scores. Such below-the-recognized quality based on DISCERN was related more to the questions 1 to 8: lacking data for the aim of the website content, authorship, relevancy, source information, and publication date. However, part of the shortcomings is attributed to the questions 9 to 15 of the DISCERN tool [7, 10]. The assessed websites contain deficient information about treatments, alternatives, and complications. Thus the lower the scores in the first two sections of the DISCERN tool, the lower the score for the final question, which simply gives a score on the overall quality of the website. The score of the first section of the DISCERN tool (questions 1–8) for a given website, could be improved easily if the authority responsible on it provided the missing information (date of post and of update, authorship, references and sources, …etc.) accurately. The same applies to the second section of the DISCERN tool (questions 9–15). To sum up, websites which achieve lower scores on DISCERN tool significantly lack adequate medical information for internet users, and introduce biases among their contents.

On the basis of JAMA benchmark tool, it is evident that the Arabic language websites on dental implants provide information below the recognized standards: the mean score was 1.69 ± 1.13 out of 4, i.e. a poor score. Such a low score on the JAMA benchmarks tool is largely attributed to e not mentioning the sources of the information, the authorship of the content, the date of posting, and the regular updating. In contrast, disclosure criterion of the JAMA benchmark tool was fulfilled by most of the included websites. With no doubts, it seems surprisingly a bad practice that medical websites contain information without fulfilling authorship, attribution and currency criteria.

Apart from the quality of the included websites which was below the recognized standards, the texts were to be found easily readable and understandable. The majority of the websites had a lower FKGL score which means they are easy to understand by an average internet user. In support of this, all included websites were found to be easy to understand by the patient with middle school background as per the SMOG results. In support of both results, the FRE score was high, indicative easy and understandable websites [8, 9]. Thus, readability understanding of Arabic information on dental implants was easy for many patients seeking online information. The low scores of JAMA and DISCERN reinforce each other that the quality of the web-based Arabic information on dental implants is low. This is supported further with a moderate proportional correlation (rho = 0.612) between them.

The study has a few limitations worth mentioning. First, we searched only in three search engines, although they are the most famous ones. We included the first 100 websites from each engine, although it is highly likely that the internet users don’t go beyond this number. There are other tools for quality assessment, other than used in our study. However, the used tools are the most frequently used in this context. The tools which used to assess the readability were developed to assess English text. However, they have been used in context of Arabic texts and reflected high validity and reliability. In future, more search engines should be included with an increased sample size across each engine to bring about results that are more accurate. Additionally, patients, their associates, and active internet users should be taken on board while developing medical-related webpages [31].

## Conclusion

Unfortunately, although easily readable and understandable, most of the easily accessible web-based Arabic health information on dental implants does not meet recognized quality standards.

## Electronic supplementary material

Below is the link to the electronic supplementary material.

Supplementary Material 1

## Data Availability

All data generated or analyzed during this study are included in this published article and the links are included in the supplementary file.

## Abbreviations

HONcode: Health on the Net Foundation Code of Conduct
JAMA: The Journal of the American Medical Association
FKGL: Flesch Kincaid grade level
ARI: Automated Readability Index
SMOG: Simple Measure of Gobbledygook
FRE: Flesch Reading Ease
SD: Standard Deviation.
