# Supplementary material for: Quality of web-based Arabic health information on dental implants: an infodemiological study
Source: BMC Oral Health. 2023 Apr 20;23:232. doi: 10.1186/s12903-023-02938-8 (PMC10116105; doi:10.1186/s12903-023-02938-8)
Supplement: Supplementary file 1 — Supplementary Material 1 [file 12903_2023_2938_MOESM1_ESM.docx]

| No. | Website |
| --- | --- |
| 1 | <https://www.mayoclinic.org/ar/tests-procedures/dental-implant-surgery/about/pac-20384622> |
| 2 | <https://ar.wikipedia.org/wiki/زراعة_الأسنان> |
| 3 | <https://tajmeeli.com/عملية-زراعة-الأسنان/> |
| 4 | <https://www.webteb.com/dental-health/treatment/زراعة-الاسنان> |
| 5 | https://www.colgate.com/ar-sa/oral-health/implants/dental-implant-surgery |
| 6 | <https://turkeywiz.com/زراعة-الاسنان/> |
| 7 | <https://www.ilajak.com/blog/dental-implants-pictures> |
| 8 | <https://www.esdent.co.uk/ar/التهاب-محيط-الغرسات-السنية/> |
| 9 | https://www.primomedico.com/ar/eilaj/lzr-lfwry-llsnn/ |
| 10 | <https://mawdoo3.com/عيوب_زراعة_الأسنان> |
| 11 | https://bimaristantr.com/specialties/dentistry/dental-implants/ |
| 12 | <https://www.marefa.org/زراعة_الأسنان> |
| 13 | https://winneresthetic.com/articles/the-process-of-dental-implants-from-a-to-z/ |
| 14 | <https://drmansour.org/زراعة-الأسنان/> |
| 15 | https://sdic.com.sa/dental-implant-jeddah/ |
| 16 | <https://wondersdentistry.com/أفضل-أنواع-زراعة-الأسنان-في-العالم/> |
| 17 | <https://veskedent.com/tedaviler/علاج-زرع-الاسنان/?lang=ar> |
| 18 | <https://arab-dental.com/ar/زراعة-الأسنان/> |
| 19 | https://emaddent.com/ar/dental-implants-blog/ |
| 20 | <https://www.dentakademi.com.tr/ar/خدمات/طب-زراعة-الأسنان/> |
| 21 | <https://www.andalusia-dentalcenters.com/زراعة-الأسنان> |
| 22 | <https://www.yetkinbayer.com/ar/تكلفة-زراعة-الاسنان.html> |
| 23 | <https://www.bokdoc.com/blog/زراعة-اسنان-ايطالية/> |
| 24 | <https://twojaws.com/الغرسة-السنية-ملف-متكامل/> |
| 25 | <https://jamilty.com/زراعة-الأسنان-أنواعها-ومميزاتها/> |
| 26 | <http://www.eurodentalcenter.com/index.php/ar/implant/528-زراعة-الاسنان-الفورية-او-المباشرة> |
| 27 | <https://medirip.com/ar/treatment/عملية-زراعة-الأسنان> |
| 28 | <https://ttafakar.com/جراحة-زراعة-الأسنان/> |
| 29 | <https://altibbi.com/مقالات-طبية/جراحة-الفك-والاسنان/اسباب-ومخاطر-جراحة-زراعة-الاسنان-5282> |
| 30 | <https://daleelturkiye.net/dental-implants-زراعة-الاسنان-في-تركيا> |
| 31 | <https://citydentar.com/معلومات/أشكال-الزرع-المختلفة> |
| 32 | <https://www.hellooha.com/articles/3066-عملية-زراعة-الأسنان-المخاطر-والتكاليف> |
| 33 | <https://www.dentspa.com.tr/ar/زراعة-الأسنان/> |
| 34 | https://www.turkeyanaclinic.com/ar/dental-implants/ |
| 35 | https://ariamedtour.com/ar/blogs/dental-implants-surgery-diet/ |
| 36 | <https://www.veraclinic.net/ar/مراحل-إجراء-عملية-زراعة-الأسنان/> |
| 37 | <https://3rabica.org/زراعة_الأسنان> |
| 38 | https://ar.stomatology.biz/xirurgiya/implantaciya/skolko-sluzhit-implant-zuba.html |
| 39 | <https://www.dw.com/ar/زراعة-الأسنان-فوائدها-ومخاطرها/a-18773526> |
| 40 | <https://medworlddental.com/ar/زراعة-الأسنان/> |
| 41 | <http://www.alajajidental.com/ar/زراعة-الأسنان/خدماتنا/> |
| 42 | <https://ethicanaclinic.com/زراعة-الأسنان-في-تركيا/> |
| 43 | https://skinandteeth.ae/dental-implants/ |
| 44 | <https://kenawydentalcenters.com/زراعة-الاسنان/> |
| 45 | <https://www.opcadental.com/ar/services/زراعة-الاسنان/> |
| 46 | https://www.bassemsamirclinics.com/services/dental-implants/ |
| 47 | <https://asnaniat.com/تجربتي-مع-زراعة-الاسنان/> |
| 48 | https://www.mouth-teeth.com/2020/04/My-experience-with-dental-implants-0.html |
| 49 | <https://capitaldental.clinic/أسعار-زراعة-الأسنان-في-مصر/> |
| 50 | <https://www.magrabi.com.sa/ar/blog/زراعة-الاسنان-بالليزر/> |
| 51 | https://www.saphiredent.com/ar/service/dental-implants-ar |
| 52 | <https://www.almhydbclinic.com/زراعة-الاسنان/> |
| 53 | <https://guaranteedental.net/زراعة-الاسنان/> |
| 54 | <https://www.micrisdental.com/ar/زراعة-الأسنان-الكل-على-٤> |
| 55 | <https://www.elconsolto.com/dental/dental-news/details/2022/1/13/2158075/زراعة-الأسنان-ضرورية-لهؤلاء-هل-لها-مخاطر-> |
| 56 | <https://teb24.news/زراعة-الأسنان-في-نابلس-كيف-تتم؟-وكم-الت> |
| 57 | <https://ridentdental.com/هل-زراعة-الاسنان-مؤلمة/> |
| 58 | <https://www.sehatok.com/أعراض-وأمراض/زراعة-الأسنان-دواعي-الإجراء-ومخاطره> |
| 59 | <https://www.babonej.com/زراعة-الأسنان-384338.html> |
| 60 | <https://medhind.com/زراعة-الأسنان-الفورية-في-الهند/> |
| 61 | <https://hyatok.com/كيف_تتم_عملية_زراعة_الأسنان؟> |
| 62 | https://www.thai-advisor.com/dental-implants-in-thailand/ |
| 63 | <https://www.drzadvisor.com/زراعة-الاسنان-في-الهند/> |
| 64 | https://www.brightway.clinic/portfolio-item/dentalimplant |
| 65 | http://nasralqudaimi.com/content.php?lng=arabic&id=22 |
